# MagneTEskin—Reconstructing skin by magnetically induced assembly of autologous microtissue cores

**DOI:** 10.1126/sciadv.abj0864

**Published:** 2021-10-08

**Authors:** Christiane Fuchs, Linh Pham, Ying Wang, William A. Farinelli, R. Rox Anderson, Joshua Tam

**Affiliations:** 1 Wellman Center for Photomedicine, Massachusetts General Hospital, Boston, MA 02114, USA.; 2 Department of Dermatology, Harvard Medical School, Boston, MA 02115, USA.

## Abstract

Skin wounds are immense medical and socioeconomic burdens, and autologous skin grafting remains the gold standard for wound repair. We recently found that full-thickness micro skin tissue columns (MSTCs) can be harvested with minimal donor site morbidity, and that MSTCs applied to wounds “randomly” (without maintaining their natural epidermal-dermal orientation) can accelerate re-epithelialization. However, despite MSTCs containing all the cellular and extracellular contents of full-thickness skin, normal dermal architecture was not restored by random MSTCs. In this study, we developed a magnetically induced assembly method to produce constructs of densely packed, oriented MSTCs that closely resemble the overall architecture of full-thickness skin to test the hypothesis that maintaining MSTCs’ orientation could further hasten healing and restore a normal dermis. Our method led to faster and more orderly re-epithelialization but unexpectedly did not improve the retention of dermal architecture, which reveals a hitherto unappreciated role for tissue morphology in determining dermal remodeling outcomes.

## Teaser

Replicating the structure and orientation of full-thickness skin by magnetically-induced assembling of micro tissue cores.

## INTRODUCTION

When biological tissue is damaged by disease or trauma beyond its capacity to regenerate, replacing the defective tissue by transplantation is often the only curative option. However, harvesting tissue for grafting creates a donor site wound. Donor site morbidity and the limited availability of donor tissue are major drawbacks for autologous grafting, while allogeneic transplants pose major risks from graft-versus-host reactions and from life-long immunosuppression. The field of tissue engineering emerged in recognition of these drawbacks, and skin was the first engineered tissue to be applied to patient care, with initial clinical use reported in the early 1980s (*1*, *2*). However, while “skin substitutes” have undoubtedly saved lives and, in some cases, enabled new therapies against previously untreatable conditions (*3*), the overall clinical utility of engineered skin has been limited, and autologous skin grafting still remains the predominant clinical option for wound repair. A major limitation of existing skin engineering technologies is their inability to faithfully replicate natural skin, a complex tissue with multiple functions and distinct microstructures, many of which are yet to be fully understood. For example, recent studies have shown that there are distinct subpopulations of dermal fibroblasts from different lineage origins that perform different functions in wound healing (*4*) and that cutaneous adipocytes are much more actively engaged in wound healing than previously recognized (*5*). While there are ongoing efforts to increase the inclusion of various skin cell types into artificial skin constructs (*6*–*8*), there is currently no engineered skin product that can incorporate all the diverse cell populations/subpopulations or reproduce the intricate extracellular architecture of natural skin. As a result of their greatly simplified composition, artificial skin products are no match for their natural counterpart at restoring the function or appearance of skin tissue. Meanwhile, skin wounds continue to be an immense medical and socioeconomic challenge, with millions of affected patients and tens of billions of dollars in medical costs annually in the United States alone (*9*). Difficulties in replicating natural tissue have beleaguered the tissue engineering field in general, such that despite many advances over the past decades, the tantalizing aspiration of replacing defective tissues with laboratory-grown versions has remained largely unfulfilled.

Instead of the conventional “bottom-up” approach of constructing engineered tissues from single cells and biomaterials, we recently explored a “top-down” strategy using small (submillimeter scale) samplings of natural full-thickness skin tissue, which we have called micro skin tissue columns (MSTCs). An important advantage of this approach is that each MSTC can be small enough that the donor site can spontaneously heal without scarring or other long-term morbidity. Because MSTCs are sampled randomly from natural skin tissue, they include all the different cellular and extracellular components of the tissue, including those that are yet to be fully characterized, and within each MSTC, all of these components retain their respective natural organization, structure, and microenvironment. In other words, rather than trying to fabricate complex multicellular tissues de novo, we are instead “copying” from nature by using as starting material, fragments of tissue in which all its normal physiologic complexities are already in place. This approach allows us to bypass the need to reproduce the microarchitecture of natural tissue (which is still the focus of most conventional tissue engineering efforts), and the main challenge becomes rather how to assemble MSTCs into constructs at the “macro” scale of natural tissues.

We previously found that applying MSTCs “randomly” (i.e., without maintaining the epidermal-dermal orientation of natural skin) into wounds substantially accelerated wound closure and reduced secondary wound contraction (*10*, *11*) and that (unlike split-thickness grafts or bioengineered skin products) MSTC treatment allowed long-term stable engraftment of most of the major epidermal and dermal cell types, including functional adnexal structures (*12*). This practical approach has been developed into a clinical wound care therapy (*13*–*15*). However, the random orientation and placement of MSTCs creates the need for MSTCs to reorganize. The various cell types must migrate to and repopulate their respective proper anatomic locations, and the time it takes to undergo this reorganization necessarily causes delays in healing a wound. It is also not yet clear whether all cutaneous cell types (particularly those associated with deeper dermal structures) are capable of undergoing such a reorganization process. The goal of this study was to assemble and implant MSTCs in a way that maintains the natural epidermal-dermal orientation of skin—epidermal heads up, dermal tails down—and to establish whether doing so could benefit the subsequent healing process in an in vivo porcine wound model.

## RESULTS

### MagneTEskin—Magnetically induced orientation and assembly of skin columns

**Fig. 1.**
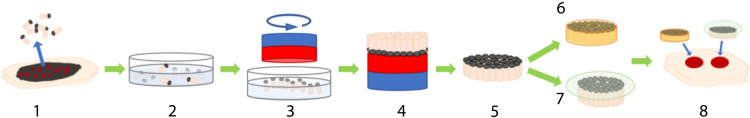
Illustration of the MagneTEskin process. (1) A magnetic coating (black layer) is applied to the epidermal surface of the donor site before harvest. (2) The harvested MSTCs are submerged in either a biomaterial solution (for the biomaterial embedding option) or sterile saline (for the topical binding option). (3) An external magnetic field is applied to induce the MSTCs to align in the same orientation and gather closely together. (4) To increase the packing density and attachment between individual MSTCs (if so desired), excess fluid could be removed by gentle dabbing with an absorbent material and letting the construct air dry. (5) The external magnetic field is removed. (6) For the biomaterial embedding option, the liquid biomaterial is induced to solidify and cross-linked to increase mechanical strength. (7) For the topical binding option, either an adhesive film dressing or a cyanoacrylate surgical glue is applied to the top surface of the assembled MSTCs. (8) The constructs are applied into wound sites.

To replicate the structure of natural skin, with the full complement of cellular and extracellular skin components all in their respective proper microanatomic locations, we sought to develop a method to satisfy the following key design criteria: (i) ability to induce and maintain the alignment of MSTCs along the epidermal-dermal orientation; (ii) assemble the aligned MSTCs into three-dimensional skin constructs resembling natural skin structure; (iii) the process should be rapid, consistent with clinical use in minutes instead of hours; (iv) the process should be scalable and customizable to fit different wound dimensions; (v) we avoided in vitro culture or other processes that would render the MSTCs more than minimally manipulated from a regulatory standpoint; (vi) minimize inclusion of exogenous materials—if any is needed, then preference will be given to Food and Drug Administration (FDA)–approved materials, and the materials must not negatively interfere with the healing process; and (vii) the final construct should be able to withstand mechanical perturbations that are typical of wound environments (manual handling, application of dressings, etc.). After testing multiple methods, we identified an effective option: By coating the donor skin with a magnetic surface layer before harvesting, the epidermal heads of each MSTC can be manipulated using an external magnetic field to control the orientation, density, and distribution of MSTCs. The coating was made by combining iron oxide (Fe_3_O_4_) particles with a commercially available silicone-based wound adhesive. Iron oxide was chosen both because of its magnetic properties and its safety profile—It is a pigment common in black tattoo inks, with a long history of tolerance by the human body, and can be removed by laser treatment if necessary. We call this method MagneTEskin—It is easily scalable and enables the assembly of large numbers of MSTCs into densely packed, properly oriented skin constructs, resembling natural full-thickness skin in both content and structure. The conceptual framework for this method is illustrated in Fig. 1.

**Fig. 2.**
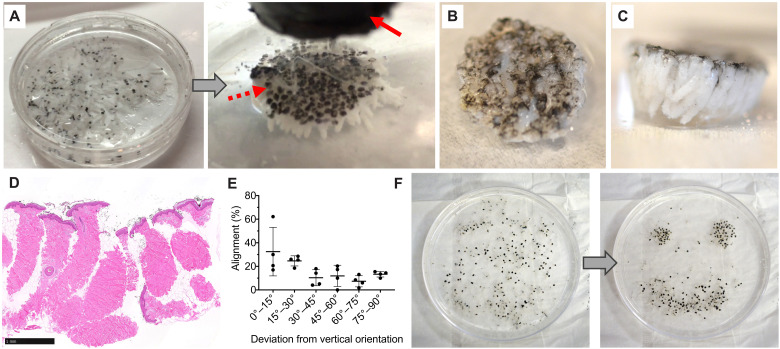
MagneTEskin method in practice. (**A**) Photographs of MSTCs before (left) and after (right) application of an external magnetic field. Dark brown coloration on epidermal heads of MSTCs was due to the magnetic coating and is highlighted by the dashed arrow. The magnet is noted by the solid arrow. Video of this process is included in the supplement. (**B**) Top view of MagneTEskin construct after the removal of the magnetic field. (**C**) Side view of the same construct showing the packing and alignment of MSTCs. (**D**) Hematoxylin and eosin (H&E) histology of an assembled construct confirming the proper packing and alignment of MSTCs. Scale bar, 1 mm. (**E**) Alignment score of ex vivo MagneTEskin constructs showing good alignment of MSTCs, with about 70% within 45° of vertical with the skin surface. (**F**) By manipulating the magnetic field, MSTCs can be arranged in specific densities and patterns. Photo credits: C. Fuchs and J. Tam, Massachusetts General Hospital.

The first requirement of the MagneTEskin method was a surface coating material with a strong magnetization response, which can remain attached to the epidermis throughout the harvesting process [when it will be subjected to various forces such as suction and fluid flow (*16*)]. This was achieved by combining iron(III) oxide particles with a silicone-based film-forming gel (Stratamed) and then applying the mixture to the donor skin surface before harvesting. This resulted in MSTCs with magnetic “tops” that could then be aligned and organized by an externally applied magnetic field (Fig. 2 and movie S1). The magnetic field could also be manipulated to produce constructs of different shapes and MSTC packing density, if so desired (Fig. 2F). To maintain the overall structure and epidermal-dermal orientation of MSTCs after withdrawal of the external magnetic field and to withstand the mechanical perturbations typically experienced by skin, the aligned MSTCs need to be bonded together in some way. To this end, a number of bonding options were investigated, which can be broadly categorized as either completely embedding the MSTCs together in biomaterials or securing the top surface of the MSTC assemblies topically with an adhesive, as depicted in Fig. 1, with details listed in Table 1. The bonding options were first evaluated for feasibility and general compatibility with the MagneTEskin assembly process and then against the design criteria described in the previous paragraph. The performances of the different assembly options against these criteria are summarized in Table 1.

**Table 1.** Performance of various assembly options against ex vivo design criteria. ✓, criterion satisfied; −, did not evaluate due to failure to pass earlier critical criteria; N/A, criterion not applicable for method in question; x, failure to meet criterion, with reason presented as table footnotes. Options highlighted in green were the ones chosen for in vivo testing. PCXL, photochemical cross-linking; CXL, chemical cross-linking.

		**Biomaterial embedding/binding of MSTCs**						**Topical protective layer**		
		Collagen	Thermoreversible hydrogel	Collagen + d-ribose	Collagen + Rose bengal (PCXL)	Collagen + EDC/NHS (CXL)	Fibrin-Collagen (1:1 ratio)	Fibrin	Film dressing	Cyanoacrylate adhesive
**Ex vivo**	Compatibility with MagneTEskin assembly method	✓	X*	✓	✓	✓	✓	✓	✓	✓
	Mechanical robustness	X^†^	__	X^†^	✓	✓	✓	✓	✓	✓
**In vivo**	Biodegradability	__	__	__	X^‡^	X^‡^	✓	N/A	N/A	N/A
	Biocompatibility	__	__	__	~	~	X^§^	✓	✓	✓
**Practicality**	Speed of production	__	__	__	Slow	Slow	Fast	Fast	Fast	Fast
	Cost of materials	__	__	__	High	High	High	High	Low	Low

*Viscosity of biomaterial impeded alignment of MSTCs.

†Construct disrupted by mechanical perturbations.

‡Substantial amounts of material failed to degrade over 8 weeks.

§Material induced exuberant eosinophilic inflammation.

### Assembly option 1: Biomaterial embedding/binding of MSTCs

The first option of embedding/binding MSTCs in a biocompatible matrix requires a biomaterial that exists initially in liquid form to allow free movement of the MSTCs. The material should then be inducible to solidify within a relatively short timeframe. The biomaterial must also be compatible with cell growth. Several biomaterials that met these criteria were chosen for testing, including neutralized bovine type I collagen, a thermoreversible hydrogel, and a 1:1 mix of collagen and fibrin. MSTCs were submerged in the biomaterial solutions and then assembled by applying an external magnetic field, and the biomaterial was caused to solidify, producing a construct of MSTCs encased in biomaterial. The thermoreversible hydrogel was quickly ruled out as the MSTCs failed to align along the magnetic field when submerged in that material. For the other materials, proper alignment of MSTCs was verified from histologic images of the MagneTEskin constructs (Fig. 2, D and E).

To test their mechanical robustness, MSTC constructs were placed into excision “wounds” in ex vivo porcine skin tissue and subjected to manual manipulation and compression, simulating the mechanical forces that the constructs would be subjected to in the in vivo setting. Collagen alone was fragile and broke apart easily with routine handling. To strengthen the collagen gel, we cross-linked the collagen with different nontoxic cross-linking reagents that have been used in other in vivo applications—specifically d-ribose, rose bengal with photocrosslinking, or *N*-(3-dimethylaminopropyl)–*N*′-ethylcarbodiimide hydrochloride (EDC) combined with *N*-hydroxysuccinimide (NHS). Details on the cross-linking parameters are described in Materials and Methods. Collagen gels cross-linked with d-ribose were still too fragile, but gels cross-linked with rose bengal or EDC/NHS, as well as collagen-fibrin gels, were all mechanically robust and were selected for further in vivo testing.

### Assembly option 2: Topical binding

A second option for securing the aligned MSTCs was by binding them only at the epidermal surface. Compared to the previous option, this second option is faster and simpler and avoids introducing exogenous materials into the body. The drawbacks are that the connections between individual MSTCs are less secure, and the loss of potential biological benefits conferred by the biomaterials. For the topical binding process, after MSTCs are oriented and assembled, they are placed into the wound site, and then a protective layer is applied to the epidermal surface. Three candidate materials were investigated for this layer: fibrin sealant, polyurethane film dressing, or cyanoacrylate surgical adhesive. All three materials performed satisfactorily in ex vivo testing of mechanical stability (as described in the previous section). Because there were no discernable differences between the different materials at this stage, the two materials that were the most inexpensive and convenient to use, namely, polyurethane film dressing and cyanoacrylate surgical glue, were chosen for in vivo evaluation.

### In vivo response to exogenous biomaterials

**Fig. 3.**
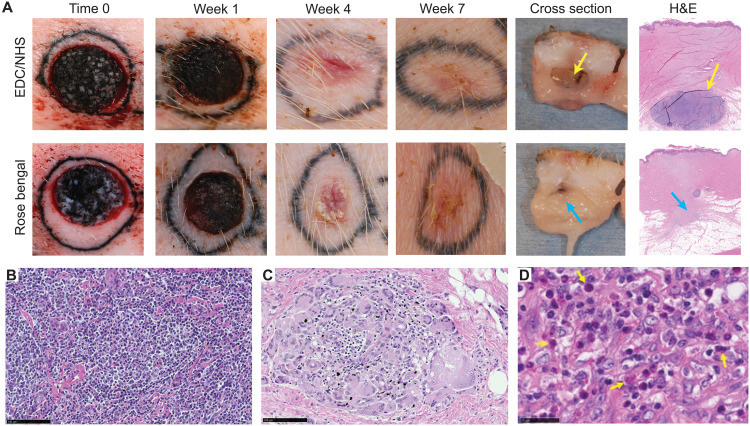
Biomaterial embedding option led to suboptimal outcomes in vivo. (**A**) Photographs at designated time points of wounds treated with MagneTEskin constructs embedded in collagen gel and cross-linked with EDC/NHS (top row) or rose bengal photocrosslinking (bottom row). Substantial wound contraction was observed in all experimental groups. Undegraded remnants of the biomaterials could still be seen visually in cross-sectioned biopsies collected at week 7 and confirmed with histology (arrows). Granulomas of various sizes were observed in both EDC/NHS (**B**) and rose bengal (**C**) cross-linked collagen biomaterial groups. (**D**) The fibrin-collagen matrices were quickly degraded but induced an exuberant eosinophilc inflammation. From H&E histology sections, infiltrating eosinophils (yellow arrows) were identified by their distinctive bilobed nuclei and intense, granular cytosolic eosin staining. Eosinophils were diffusively present throughout the wound beds in wounds treated with the collagen-fibrin constructs (1-week sample shown) but were not found in any other treatment/control groups. Scale bars, (B and C) 100 μm and (D) 25 μm. Photo credit: C. Fuchs, Massachusetts General Hospital.

The various MSTC assembly options chosen from ex vivo testing were evaluated in the porcine full-thickness excision wound model, because the pig is, by far, the most faithful model for human wound healing (*17*). The wounds were monitored for 8 weeks after implantation to gauge effects on healing and tissue remodeling. For constructs of MSTCs embedded in biomaterial matrices, the collagen gels cross-linked by either EDC/NHS or rose bengal were slow to degrade, with remnants of the material persisting in the wound sites, accompanied by foreign body reactions (Fig. 3, A to C). The collagen-fibrin gels did degrade within the re-epithelialization timeframe but elicited an exuberant eosinophilic inflammatory reaction (Fig. 3D). The treated wounds also showed substantial secondary contraction and loss of normal dermal collagen architecture. These results, together with the more complex and costly production process, led us to conclude that the biomaterials evaluated in this study were suboptimal for producing MagneTEskin constructs.

### Topical binding: Improved organization and faster re-epithelialization with aligned MSTCs

**Fig. 4.**
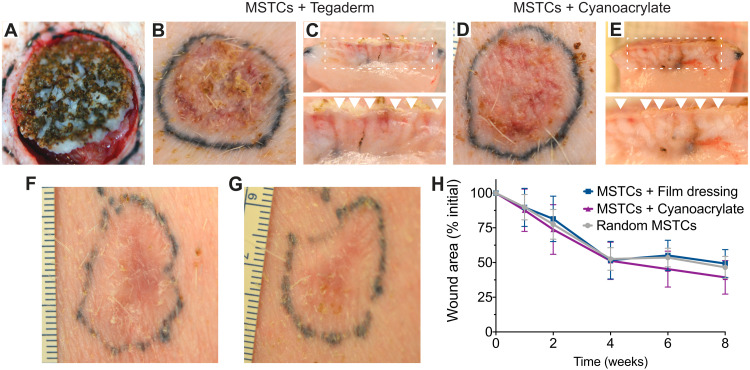
MagneTEskin constructs with topical binding in vivo. (**A**) MagneTEskin construct immediately after placement into a wound. (**B** to **E**) Representative photographs of top (B and D) and cross-sectional (C and E) views of MagneTEskin constructs at 2 weeks after treatment. (C and E) Aligned MSTCs are observable in the cross sections (denoted by arrow heads in the bottom images, which are higher-powered views of the wound beds, specifically the regions highlighted by dotted lines in the top images). Representative photographs of MagneTEskin and control wounds at 8 weeks are shown in (**F**) and (**G**), respectively. Edge of images, (F and G) 3 cm. (**H**) Wound contraction over time was similar between MagneTEskin and random MSTC groups. Photo credits: C. Fuchs and J. Tam, Massachusetts General Hospital.

**Fig. 5.**
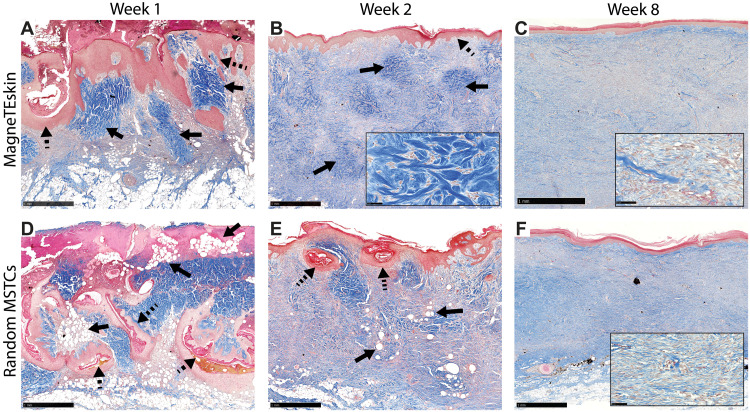
MagneTEskin histology. Trichrome-stained histology images of the designated experimental groups and time points. (**A**) The alignment and organization of MagneTEskin constructs was apparent at week 1, with dermal (blue, highlighted by solid arrows) and epidermal (red, highlighted by dashed arrows) elements positioned appropriately at their respective microanatomic locations, and adipocytes mostly confined to the subcutaneous space. The iron oxide coating largely remained at the epidermal surface and was rarely found inside the wound beds. (**D**) Random application of the same amount of MSTCs resulted in a chaotic architecture at week 1, with epidermal fragments buried deeply under the wound surface (dashed arrows), and adipocytes throughout the wound bed as well as the wound surface (solid arrows). (**B**) By week 2, MagneTEskin-treated wounds had complete, contiguous epidermal coverage (dashed arrow). The crisscrossing collagen fiber architecture of normal dermis still remained (solid arrows, high-powered view shown in inset) but appeared to be fading compared to week 1. (**E**) In the random MSTC group, tissue remodeling had also progressed by week 2—The epidermis was mostly located at the wound surface, with occasional epidermal remnants in the dermal regions appearing to be migrating upward (dashed arrows) and fewer intradermal adipocytes compared to week 1 (solid arrows). (**C**) MagneTEskin construct at week 8. The dermal collagen at this point mostly consisted of fine fibrils arranged in parallel to the skin surface, and the overall architecture was similar to the corresponding random MSTC controls (**F**). Higher-powered views shown in inset. Scale bars, (main figures) 1 mm and (insets) 50 μm.

The topically bound MagneTEskin constructs were first evaluated in the short term, specifically the initial 1 to 2 weeks after implantation, for their ability to maintain overall structure and MSTC alignment over this period, because this assembly option was thought to be less secure, and in our experience, wound implants are most vulnerable to mechanical disruptions during the first 2 weeks. Similar amounts of MSTCs implanted randomly into similar wounds were used as controls. In the topically bound MagneTEskin group, the successful maintenance of MSTC alignment under in vivo conditions was visually apparent in biopsy samples (Fig. 4, B to E) and confirmed by histology (Fig. 5): At 1 week after implant, the MSTCs’ epidermal heads were appropriately located at the skin surface, dermal portions of the wounds were filled with dermal tissue from the MSTCs, and adipocytes were properly located in the subcutaneous space (Fig. 5A). In stark contrast, wounds treated with similar amounts of “random” MSTCs had a chaotic organization, with epidermal fragments and adipocytes haphazardly scattered throughout the wound bed, and the formation of keratotic cysts (Fig. 5D). By week 2, the MagneTEskin groups succeeded in completely re-epithelializing the wounds with well-formed, contiguous, fully stratified epidermal coverage (Fig. 5B), whereas in the control group, there was still on-going epidermal reorganization and subepidermal cysts, albeit to a lesser extent than at the 1-week time point (Fig. 5E).

### Dermal degradation and reorganization still occurred in oriented MSTCs

Given the encouraging short-term healing results, MagneTEskin constructs with topical binding were evaluated for long-term outcomes, particularly whether the dermal collagen architecture could be retained [as it is in full-thickness skin grafts (FTSGs)]. Compared with untreated wounds, the MagneTEskin constructs significantly reduced the time for wound healing and reduced wound contraction. The MSTCs’ normal dermal architecture appeared intact at week 1 and was still present but slightly fading by week 2. By 4 weeks after implantation, the crisscrossing basket-weave collagen architecture could no longer be seen; instead, the dermis was occupied by thin collagen fibers largely oriented in parallel with the skin surface. This disrupted architecture continued into the eighth week (Fig. 5C). Mirroring these histologic findings, wounds treated with oriented MSTC constructs showed similar contraction as wounds treated with random MSTCs (Fig. 4, F to H). Thus, we found that assembling MSTCs into densely packed constructs with proper epidermal-dermal orientation enabled a more orderly remodeling process and earlier restoration of the epidermis. However, the assembled MSTCs did not lead to long-term preservation of the dermal architecture. Implications of these findings are discussed below.

## DISCUSSION

In this study, we established a practical method to manipulate and organize large numbers of tissue cores into defined structures and patterns and showed that this method could be used to produce constructs of densely packed, oriented MSTCs that closely resemble the natural architecture of full-thickness skin. The ability to manipulate and assemble small “building blocks” into larger tissue constructs has long been recognized as a key capability need toward enabling the engineering of complex solid tissues, as evidenced by the many different approaches that have been developed over the years aiming to achieve this goal (*18*). Most previous efforts have been tailored for building blocks composed of laboratory-fabricated cell-biomaterial combinations, whereas ours is a method developed for manipulating small units of natural living tissue. This method should be applicable to multiple tissue types, because most tissues are capable of regeneration after small injuries (where the definition of “small” is likely to be tissue specific), and the magnetic material used in our approach, namely, iron oxide, has been tolerated by the human body for millennia in the form of tattoo ink. Our method could be used to orient tissue fragments from essentially any tissue with an accessible epithelium to which a paramagnetic material coating can be applied. Moreover, magnetic fields could be used for more complex tissue orientation. We used a large, simple permanent magnet to orient and align the epidermal surface of skin MSTCs. An array of small electromagnets could potentially be used to selectively move tissue components during construction of engineered tissues.

We also showed that keeping MSTCs in proper orientation resulted in improved organization and faster completion of the re-epithelialization process, compared to equivalent amounts of randomly applied MSTCs [the latter is itself superior to wound closure by secondary intention, as we previously established (*10*, *11*)]. The advantage of MSTC orientation is temporary in our acute wound model, as keratinocytes from the randomly applied MSTCs were eventually able to migrate to the skin surface and reorganize to form a contiguous epidermis and spontaneously resolve the problems caused by the initial ectopic location of keratinocytes, such as epidermal cysts. This speaks to the extraordinary ability of keratinocytes to migrate substantial distances through wound tissue toward the tissue-air interphase and reorganize there to form an epidermis, so long as a moist wound environment is maintained, as has been reported previously in randomly placed minced skin or skin grafts that were intentionally implanted upside down (*19*–*21*). The advantages in quicker and more orderly re-epithelialization that come with maintaining the epidermal-dermal orientation of MSTCs, compared to applying MSTCs randomly into wound beds, come at the cost of a more complex, costly, and time-consuming assembly process, as well as requiring much larger amounts of MSTCs—Our previous work with the random approach used about 20% mass replacement, while at least 80% is needed when MSTCs are oriented and tightly packed without biomaterials. For most clinical applications, the cost-benefit balance is likely to favor the random approach. A potential exception could be cases where the ability of keratinocytes to migrate and reorganize is compromised (e.g., due to disease or aging), such that the benefits to keeping MSTCs oriented could be more substantial.

**Fig. 6.**
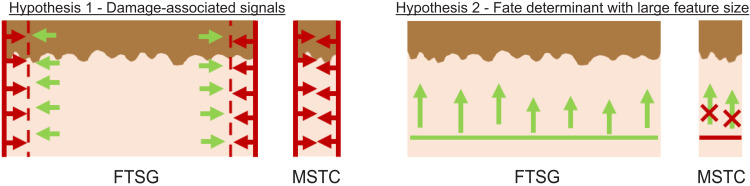
Two hypotheses for the divergent tissue fates between FTSGs and MSTCs. The epidermis is depicted with light brown color at the top, and the dermis is depicted in flesh color at the bottom. Because FTSGs and MSTCs are similar in content and microanatomy, the determinant factor(s) are likely related to graft morphology. In the first hypothesis (left), damage-associated signals (red arrows) are released from the cut edges of the graft (solid red lines). In certain regions adjacent to the cut edge, these damage signals are dominant (dashed red lines), resulting in degeneration of dermal architecture. In FTSGs, these degenerative regions are relatively small compared to the intact areas of the graft where homeostatic signals for maintaining the dermal architecture (green arrows) are dominant. In MSTCs however, because of the high ratio between cut surface area and graft volume, the degenerative region may dominate the entire graft. In the second hypothesis (right), the critical (likely structural) factors (solid green line) that provide the homeostatic signals (green arrows) have feature sizes much greater than the dimensions of MSTCs, such that when these factors are disrupted by MSTC harvesting, the homeostatic signals are lost and the dermal architecture degenerates during tissue turnover.

Conceptually, the demand for source tissue could be partially alleviated by arranging MSTCs at lower densities and using biomaterials to fill any void space as well as support the oriented MSTCs. As shown in Fig. 2F, the MagneTEskin method does allow some flexibility over both MSTC packing density and distribution, and while the biomaterial formulations tested in this study all had drawbacks, given the vast variety of biomaterials that have been developed for different biomedical applications, it seems likely that, with more exhaustive screening, a suitable biomaterial (ideally one that could prevent the dermal degradation process seen in this study) could be identified/formulated if so desired. For the current study, we elected to focus on assembling MSTCs at high packing densities to mimic FTSGs by maximizing the presence of natural skin tissue in the wound volume, with the goal of restoring and maintaining the natural dermal architecture—the “holy grail” that has eluded the skin tissue engineering field for decades. The difference in dermal matrix remodeling between FTSGs (containing full-thickness skin including the entire depth of the dermis) and split-thickness skin grafts (STSGs; containing only the epidermis and the upper portion of the dermis), where the former is able to retain the normal dermal matrix architecture, while the latter degenerates into fibrotic scars, suggests that there exist certain deep dermal elements (as yet unidentified) that are critical to the preservation of dermal architecture. Both FTSGs and STSGs exhibit a similarly elevated rate of collagen turnover after grafting, leading to almost complete replacement of dermal collagen within roughly 2 months in animal models (*22*). However, while in STSGs, the degradation of collagen exceeded its production rate, leading to a net loss of collagen, in FTSGs, production exceeded degradation, resulting in a net gain (*23*). This implies that the preservation of dermal architecture in FTSGs is not just a static retention of the grafted material, but rather it is due to an active process and/or microenvironmental factor(s) in FTSGs that enables the dermal remodeling to proceed in such a way as to retain the normal microanatomy, while the lack of similar cues in STSGs causes the dermal matrix to degrade and be replaced by fibrotic scarring. Because MSTCs collectively contain all the same cellular and extracellular components as FTSGs, and these components were located at the appropriate skin depths in the oriented, we reasoned that they may similarly be able to preserve and restore the dermal matrix. However, contrary to our initial expectation, maintaining the orientation of MSTCs did not improve the long-term retention of the dermal matrix, at least within the study timeframe. Notably, the presence of normal dermal architecture in the MSTCs at early (1 to 2 weeks) time points suggests that its subsequent disruption was most likely caused by an active degradative process, possibly triggered by the harvesting procedure, and/or differences in morphologic characteristic between MSTCs and FTSGs. We have two main hypotheses for the key difference between MSTCs and FTSGs (Fig. 6). First is the local injury response to cutting at the graft edges, which is known to cause the release/activation of many damage-related signals (*24*–*27*). MSTCs have a much higher ratio of cut edge surface area to tissue volume, which may cause the damage-associated signals from the cut edges to overwhelm the normal homeostatic tissue maintenance mechanisms. The MSTC size (~700 μm diameter) chosen for this study was based on our previous finding that donor site scarring can be avoided at this harvest size (*10*). Future studies characterizing the size threshold that triggers the degenerative versus maintenance response (as occurs in FTSGs) in the graft tissue should provide valuable information both for continued research attempts to restore normal full-thickness skin and for making informed clinical decisions balancing donor site scarring against dermal tissue preservation. Our second hypothesis is that factors responsible for maintaining the dermal architecture may have feature sizes that exceed the dimensions of individual MSTCs, such that they are rendered ineffective by the harvesting procedure. Structural components of the skin, such as collagen and elastin networks, seem more likely for this role, and changes in mechanical properties when these extracellular networks are disrupted may be a contributing factor, as mechanotransduction pathways are known to be key mediators influencing long-term scarring versus regeneration in skin wounds (*28*). We have previously shown that MSTCs exhibited an altered paracrine production profile compared to FTSGs (*29*), and the results from the current study suggest that there are additional tissue-level effects related to the harvesting and/or morphology of MSTCs that are yet to be fully understood. Deciphering the underlying mechanism(s) governing this morphology dependency of the ability to preserve normal dermal architecture could be a critical step toward engineering physiologically normal full-thickness skin tissue.

## MATERIALS AND METHODS

### Animal model

All animal procedures were performed in accordance with the Public Health Service Policy on Humane Care and Use of Laboratory Animals and with approval by the Massachusetts General Hospital Institutional Animal Care and Use Committee. Adult female Yorkshire swine, around 40 kg at time 0, were used for this study. Autologous full-thickness MSTCs were collected using a custom-made, suction-assisted harvesting device with 19-gauge harvesting needles, as described previously (*10*, *16*). For the wound model, a series of 1.5-cm-diameter, full-thickness skin wounds were produced on the trunk skin of the animals by excision down to subcutaneous fat. The excised tissue was weighed, and the wound margins were tattooed to facilitate subsequent identification. Constructs with MSTCs aligned in the natural epidermal-dermal orientation were produced using our MagneTEskin approach, as detailed below. Wounds treated with identical amounts of MSTCs randomly spread over the wound bed served as controls. To avoid potential confounding effects of having increased amounts of the magnetic coating material buried deep inside the wound beds, control MSTCs were not coated with the magnetic coating. Treatment and control wounds were placed in alternating spatial patterns to account for anatomical variations in skin properties. All wounds were dressed by a combination of hydrogel and foam dressings (Tegaderm, 3M Health Care, St. Paul, MN), as previously shown to enable keratinocyte reorganization after randomized application of minced split-thickness skin fragments (*21*). The dressed wounds were additionally protected by an adherent dressing (DuoDERM CGF, ConvaTec, Greensboro, NC), followed by stockinette and a nylon jacket (Lomir Biomedical, Malone, NY). At least four independent wound sites from two different animals were evaluated for each treatment/control group at each time point. Wound dressings were changed at least weekly until the wounds were visibly closed, and the wounds were monitored and photographed regularly for up to 8 weeks. At designated time points, wound samples were excised and preserved for histology.

A ruler was included with each wound photograph for scale. The wound edge (defined for this study as the border with visibly normal skin, where wound tissue is distinguishable due to differences in color, texture, or lack of epithelium) in each photograph was outlined by two independent evaluators who were blinded to the experimental group assignments. The outlined areas were quantified from the photographs using Fiji (*30*), and averaged values between the two evaluators were used to determine the extent of wound contraction in the final analysis.

### MagneTEskin—Magnetically induced assembly of MSTCs

To enable MSTCs to respond to external magnetic fields, a coating was produced by thoroughly mixing iron oxide particles [iron(III) oxide Fe_3_O_4_, nanopowder, <50 nm, MilliporeSigma] with a silicone-based film-forming dressing (Stratamed, Stratpharma, Switzerland) at a 2:1 (w/w) ratio. This coating was applied in a thin layer over the donor tissue surface and then allowed to dry and set for about 30 to 45 min, followed by MSTC harvest as described above. About 0.8 g of MSTCs, which corresponded to roughly 80% the weight of the excised tissue (and the maximum packing density of reassembled MSTCs based on preliminary studies), were placed into individual petri dishes filled with either a biomaterial solution (for the embedding assembly option, detailed below) or sterile saline (for the topical binding option). For the embedding assembly option, candidate biomaterials were chosen on the basis of physical characteristics (specifically, our method requires the biomaterials to initially be in the liquid state and then inducible to solidify within a relatively short timeframe), compatibility with cell growth, existence of FDA-approved versions of the material, and previous utility for engineering skin tissue. Biomaterials tested included neutralized bovine type I collagen (5 mg/ml; PureCol EZ, Advanced BioMatrix), fibrin surgical adhesive (Evicel, Ethicon), thermoreversible hydrogel (Mebiol, Advanced BioMatrix), and a 1:1 mix of collagen and fibrin [similar to mixtures of collagen and fibrin that have been previously used for skin tissue engineering (*31*)]. Neodymium magnets (McMaster-Carr) with diameters of 1.5 cm were disinfected by sequential soaking in iodine and ethanol and then brought close to the MSTCs. The iron oxide coating is attracted to the neodymium magnet, causing alignment of the MSTCs in the process (Fig. 2A). At this point, the process diverged for the two MagneTEskin assembly options (biomaterial binding versus topical binding). For the embedding option, the biomaterial-infused constructs were induced to solidify by incubation at 37°C (for collagen) or enzymatic induction (for fibrin). To improve mechanical robustness, the collagen-based constructs were additionally cross-linked with EDC/NHS (EDC and NHS at 16.5 and 3 mM for the high concentration option and 6.6 and 1.2 mM for the lower concentration option, respectively) or rose bengal (10 μM rose bengal photocrosslinked by 150 or 100 J/cm^2^ irradiation from a KTP (potassium titanyl phosphate) solid-state laser, OcuLight OR, IRIDEX). These cross-linking options were selected on the basis of prior utilization in regenerative medicine (rose bengal, particularly, is an FDA-approved reagent) (*32*, *33*). EDC, NHS, and rose bengal were all purchased from MilliporeSigma. There was no discernable difference between the two EDC/NHS concentrations, or the two laser irradiation settings; therefore, results from those trials are presented in aggregate in the manuscript.

For the topical binding option, the densely packed, oriented MSTC constructs were dabbed lightly on sterile gauze and then left to dry further for at least 30 min to increase adhesion between individual MSTCs. The constructs were then gently lifted from the magnets with nonmagnetic forceps or surgical rulers and implanted into the recipient excision wounds. The surface of the construct was further protected by applying either a polyurethane film dressing (Tegaderm, 3M) or a thin layer of cyanoacrylate surgical adhesive (Dermabond, Ethicon).

To verify the robustness of the various MagneTEskin constructs against mechanical perturbations, the constructs were placed into excision wounds in ex vivo porcine skin tissue and subjected to manual manipulation and pressure to simulate the mechanical forces that the constructs would be subjected to during standard handling and grafting procedures. Only constructs that were able to retain the MSTC constructs’ alignment and overall structure were selected for in vivo evaluation.

### Histology

Tissue samples were fixed in 10% formalin for about 48 hours and embedded in paraffin. Sections (5 μm) were stained with hematoxylin and eosin for general histology and Gomori’s trichrome for collagen. Stained slides were scanned and saved in digital format using the NanoZoomer (Hamamatsu, Bridgewater, NJ). To evaluate the alignment of MSTCs, histologic images of the MagneTEskin constructs were loaded into Fiji (*30*), then the mid-line axis of each MSTC was drawn up to the stratum corneum, and a second line was draw parallel to the skin surface. The angle between these two lines was then taken as the alignment angle of that individual MSTC.

### Statistics

For statistical analysis, each wound site was considered an independent sample. Wound contraction data were analyzed using GraphPad Prism version 9.0.1 for macOS (GraphPad software, San Diego, CA). Comparisons were made using the two-tailed Student’s *t* test (for two groups). *P* values ≤ 0.05 were considered statistically significant. Results are plotted as means ± SE.

## Notes

View/request a protocol for this paper from *Bio-protocol*.
