# Supplementary material for: MagneTEskin—Reconstructing skin by magnetically induced assembly of autologous microtissue cores
Source: Sci Adv. 2021 Oct 8;7(41):eabj0864. doi: 10.1126/sciadv.abj0864 (PMC8500515; doi:10.1126/sciadv.abj0864)
Supplement: Supplementary file 1 — Movie S1 [file sciadv.abj0864_sm.pdf]

Science Advances
AAAS

Supplementary Materials for

MagneTEskin—Reconstructing skin by magnetically induced assembly of autologous microtissue cores

Christiane Fuchs, Linh Pham, Ying Wang, William A. Farinelli, R. Rox Anderson, Joshua Tam*

*Corresponding author. Email: jtam3@mgh.harvard.edu

Published 8 October 2021, *Sci. Adv.* **7**, eabj0864 (2021)
DOI: 10.1126/sciadv.abj0864

Other Supplementary Material for this manuscript includes the following:

Movie S1
